# Cell–Matrix Interactions in Renal Fibrosis

**DOI:** 10.3390/kidneydial2040055

**Published:** 2022-12-07

**Authors:** Kristin P. Kim, Caitlin E. Williams, Christopher A. Lemmon

**Affiliations:** Department of Biomedical Engineering, Virginia Commonwealth University, Richmond, VA 23284, USA

**Keywords:** renal fibrosis, extracellular matrix, cell–matrix interactions, cell signaling pathways, fibronectin, collagen, fibroblasts, epithelial cells

## Abstract

Renal fibrosis is a hallmark of end-stage chronic kidney disease. It is characterized by increased accumulation of extracellular matrix (ECM), which disrupts cellular organization and function within the kidney. Here, we review the bi-directional interactions between cells and the ECM that drive renal fibrosis. We will discuss the cells involved in renal fibrosis, changes that occur in the ECM, the interactions between renal cells and the surrounding fibrotic microenvironment, and signal transduction pathways that are misregulated as fibrosis proceeds. Understanding the underlying mechanisms of cell–ECM crosstalk will identify novel targets to better identify and treat renal fibrosis and associated renal disease.

## 1. Introduction

Chronic kidney disease (CKD) is estimated to affect approximately 11 to 13% of the global population [1]. Progression of CKD is affected by a number of conditions such as age, chronic inflammation, diabetes, autoimmune disorders, and severe infection in the kidney [2]. One of the final pathological outcomes of CKD is renal fibrosis [2,3]. Renal fibrosis is the result of excessive accumulation of extracellular matrix (ECM) that disrupts renal function [2,4–6]. Some of the major hallmarks of long-term fibrosis include tubular atrophy, tubular dilation, increased fibrogenesis, and increased scar formation [1,3]. Renal fibrosis can be categorized based on the affected renal structures: fibrosis in the glomerulus is referred to as glomerulosclerosis; fibrosis in the proximal and distal tubules is referred to as tubulointerstitial fibrosis; and fibrosis around the vasculature is referred to as perivascular fibrosis [7]. Common diagnostics of CKD and renal fibrosis rely on end-stage markers of renal failure, measured by decreased glomerular filtration (eGFR) and increased proteinuria (albumin concentration in the urine), signifying a loss of transport capabilities and disruptions in the glomerular filtration barrier [4,8]. Although the mechanisms that initiate fibrosis are essential for tissue repair, prolonged activation of these mechanisms leads to chronic fibrosis and renal failure. Current research has focused on identifying biomarkers that can identify and treat renal fibrosis prior to end-stage renal failure.

The primary feature of renal fibrosis is the excess deposition and assembly of ECM. This increased ECM deposition causes changes in both the chemical and mechanical environments within the tissue, altering cellular function and exacerbating renal fibrosis. Notably, remodeling in the basement membrane and interstitial space encourage malfunction of the renal system [5]. ECM proteins are assembled into scaffold-like structures by the surrounding renal cells. In turn, cells bind to this de novo ECM, inducing altered signal transduction and cell behavior, that contributes to this exacerbated ECM assembly [2,5]. As such, understanding the bi-directional interactions between renal cells and the ECM is essential to identify therapeutic approaches to disrupt this cycle. In this review, we will discuss advances in understanding of interactions between renal cells and their surrounding microenvironment that drive the progression of renal fibrosis. We will highlight major ECM-secreting and assembling cells in the kidney implicated in fibrosis; identify changes in the renal microenvironment during fibrosis that drive subsequent phenotypic changes; discuss the physical interactions between cells and ECM in the renal microenvironment; and finally, discuss relevant cell signal transduction pathways that are disrupted during renal fibrosis. Together, this review will highlight cell–ECM interactions that could serve as therapeutic targets to disrupt the cyclical nature of renal fibrosis.

## 2. Cells Involved in Renal Fibrosis

**Figure 1.**
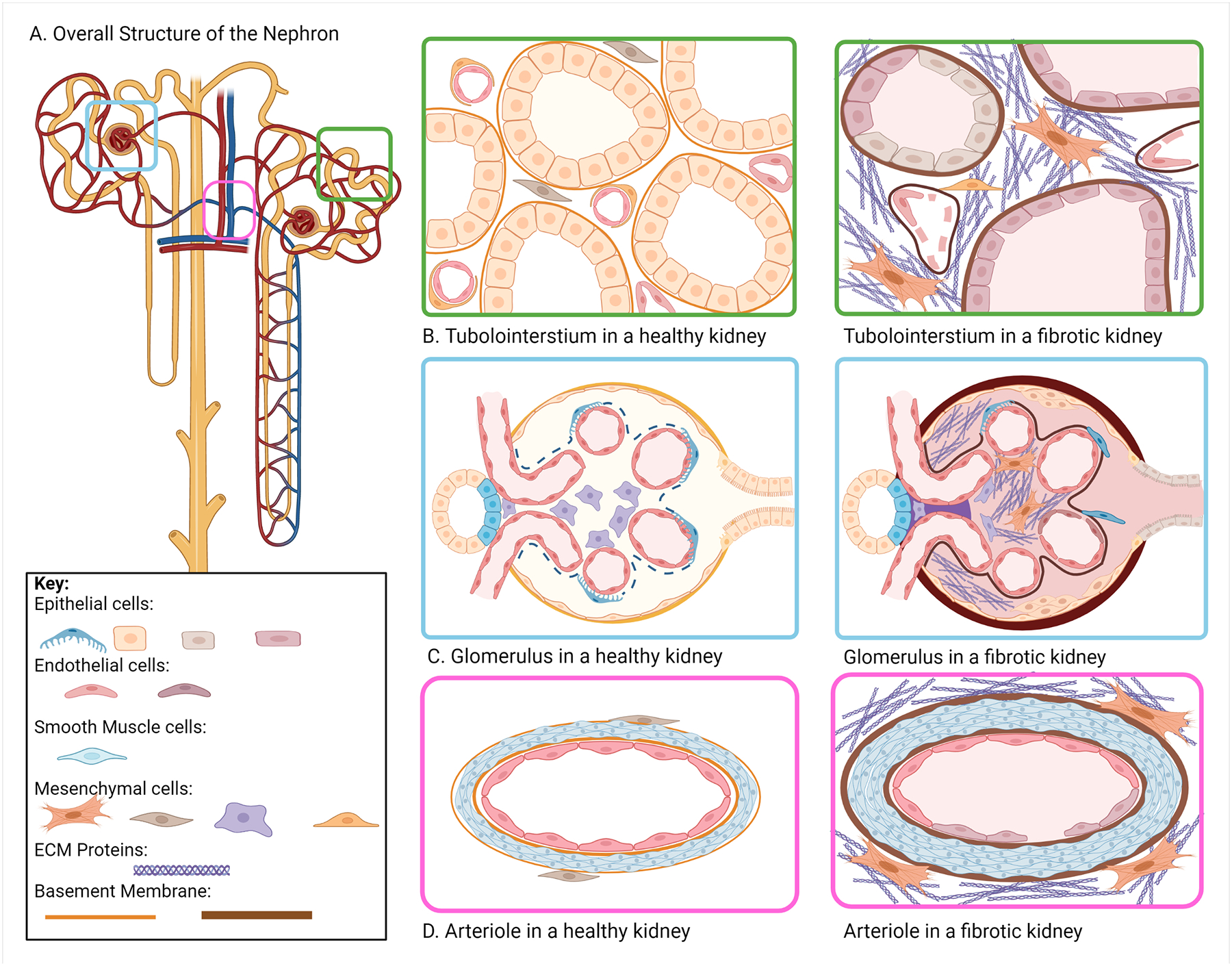
Cell–Matrix Interactions in the Healthy and Fibrotic Kidney. Healthy kidney function is driven by interactions between cells and the surrounding ECM making up the nephrons. Changes in overall ECM composition and organization disrupt these interactions and develop a profibrotic environment. (A) The overall structure of the nephron; (B) In the tubulointerstitium, tubular epithelial cells and vascular endothelial cells are surrounded by a thin basement membrane, with renal fibroblasts and pericytes encapsulated in the matrix or surrounding the vasculature, respectively. During renal fibrosis, the basement membrane thickens, the tubule dilates and thins, and renal fibroblasts and pericytes drive excess ECM assembly; (C) In the glomerulus, glomerular capillaries are encapsulated in the epithelial cells of Bowman’s Capsule. Renal fibrosis promotes the assembly of ECM within the glomerulus, thickening of the vascular basement membrane, and thickening of the Bowman’s Capsule basement membrane; (D) Perivascular sclerosis is characterized by increased smooth muscle cell proliferation and excessive ECM assembly resulting in thicker basement membranes surrounding the vasculature. Figure generated using Biorender.com.

The kidney functions as the main site of nutrient exchange and waste removal in the body, relying heavily on its complex structure to maintain homeostasis. Three distinct compartments make up the nephron, the functional unit of the kidney. The glomerulus is involved in filtering nutrients and waste from blood, the tubulointerstitium regulates transport and nutrient exchange, and the vasculature transports blood to and from the kidney [7]. Long-term progression can impact all structures of the kidney, affecting the function of specialized cells in each compartment (Figure 1). In this section we will discuss the prominent cell types that contribute to renal fibrosis through direct and indirect promotion of ECM deposition.

### 2.1. Renal Pericytes and Fibroblasts

Renal pericytes and renal fibroblasts are mesenchymal cells that play key roles in maintaining the physiological structure of the kidney. Renal fibroblasts are key producers of ECM in the tubulointerstitium and glomerulus. They are essential in providing structural and mechanical support to the kidney by maintaining the basement membrane surrounding the tubules and vasculature [9]. Pericytes are crucial in the development and stabilization of the vascular network, covering 10 to 50 % of the entire surface [10,10–12]. They regulate oxygen transport by producing renal hormones renin and erythropoietin (EPO) and are characterized through their increased expression of platelet-derived growth factor receptor-*β* (PDGFR*β*) [9,10]. In the glomerulus, mesangial cells are a specialized form of pericytes found in the juxtaglomerular compartment that interact with surrounding endothelial cells and podocytes to regulate glomerular filtration in response to vascular stretch [12].

Previous work using single-cell RNA sequencing has shown that renal fibroblasts and pericytes are the primary source of myofibroblasts in renal fibrosis [11,13,14]. Myofibroblasts are the main promoter of fibrotic progression, exhibiting a pro-migratory phenotype through increased expression of *α*-Smooth Muscle Actin (*α*-SMA) and deposition of ECM components including Col, fibronectin, and glycosaminoglycans to repair injured tissue [9,11,12]. After the injured tissue has been repaired, differentiated myofibroblasts undergo apoptosis and decrease inflammatory signals in the repaired tissue. However, in a fibrotic environment, these myofibroblasts fail to undergo apoptosis and continue secreting ECM components and remodeling the surrounding microenvironment [15]. Chronic inflammation also leads to continued differentiation of progenitors into new myofibroblasts, increasing the damage to the tissue [9]. During fibrosis, pericytes exhibit increased myofibroblast markers, including increased expression of *α*-SMA, upregulated Col deposition, and increased migration away from the vasculature [13,16,25]. This active pericyte differentiation results in their detachment from the vasculature [12,26]. Along with a loss of vascular stability, the decreased pericyte population drives a reduction in the secretion of both renin and EPO that impairs blood flow through the renal vasculature [17]. In addition to fibroblasts and pericytes, bone marrow-derived fibroblasts are also a proposed source of myofibroblast population in fibrotic diseases; however, their contribution is not fully understood [13].

### 2.2. Epithelial and Endothelial Cells

Renal structure and function are regulated by specialized epithelial and endothelial cells. A single layer of epithelial cells known as podocytes form the Bowman’s Capsule in the glomerulus, and are continguous with tubular epithelial cells (TECs) that form the Loop of Henle and the collecting duct [7]. Endothelial cells line the vasculature from the afferent arterioles and form the capillary structures within the glomerulus, at which point they merge to form the lining of the efferent arterioles and maintain the glomerular filtration barrier [7].

Epithelial and endothelial cells differentiate into a mesenchymal phenotype through the process of epithelial-mesenchymal transition (EMT) or endothelial-mesenchymal transition (EndoMT), respectively [18,19]. EMT and EndoMT are transdifferentiation processes where cells lose key phenotypic markers such as strong cell–cell adhesions, apicobasal polarity, and a cobblestone morphology, and acquire mesenchymal characteristics [20]. Although EMT has been implicated in in vitro research, there are few in vivo studies that have shown a major contribution of epithelial or endothelial cells to the myofibroblast population [11,21] in renal fibrosis. Instead, renal epithelial and endothelial cells are thought to undergo partial EMT, where they express both epithelial and mesenchymal markers without losing their cobblestone morphology, cell–cell contacts, or tubular epithelial structure [21,22]. Instead of acting as direct myofibroblast progenitors like fibroblasts and pericytes, these cells play major roles in directing myofibroblast differentiation and maintaining activity during fibrotic progression through cytokine secretion and excess secretion of ECM proteins [18]. This is in contrast to other pathological events, such as tumor metastasis, where epithelial cells undergo full EMT to promote cancer cell migration and invasion [23,24].

As stated before, pericytes play an important role in healthy tissue in stabilizing the vasculature [25]. However, when the kidney is injured, pericytes will transdifferentiate into myofibroblasts, and detach from the vasculature. This migration away from the vasculature leaves the endothelial cells vulnerable to further injury that leads to vascular rarefaction and injury to surrounding tubules [16]. Increased TGF-*β* signaling and cytokine interaction also lead to higher rates of apoptosis of endothelial cells, causing further degradation that leads to the full dissolution of the vessels [26].

### 2.3. Immune Cells

One of the key diagnostic criteria of renal fibrosis is decreased eGFR, signifying damage to the glomerular filtration barrier. Increased damage to the barrier promotes the infiltration of immune cells such as monocytes and lymphocytes [27]. Monocytes differentiate into macrophages to promote repair in injured tissue [27]. In acute healing, macrophages change from a pro-inflammatory (M1) to anti-inflammatory (M2) phenotype [28]. However, fibrosis promotes increased pro-inflammatory macrophage activity including production of reactive oxygen species (ROS), inflammatory cytokine secretion, and synthesis of pro-fibrotic matrix metalloproteinases (MMPs) [27,29]. The release of inflammatory signals induces changes in surrounding cell morphology such as myofibroblast differentiation, EMT, and EndoMT [29]. Mediation of M1 polarization of macrophages in injured kidneys reduces the release of inflammatory cytokines and excessive ECM deposition [30]. Along with M1 polarization, treatment of UUO (unilateral ureteral obstruction) mice with the anti-inflammatory quercetin downregulated M2 macrophage polarization in the injured tubulointerstitium [30], suggesting that targeting overall macrophage infiltration and polarization in the kidney may ameliorate chronic inflammation associated with renal fibrosis.

Macrophages have also recently been proposed as a source of the myofibroblast population in cases of progressive fibrosis and end-stage renal failure [28]. Increased bone-marrow derived macrophages are present in fibrotic kidneys and exhibit increased co-expression of macrophage markers and myofibroblast markers such *α*-SMA [30]. The depletion of macrophages in glomerulonephritis has been shown to reduce immune complex-mediated glomerulonephritis and reduce fibrotic progression [28]. Given that macrophage accumulation and macrophage–mesenchymal transition (MMT) promote fibrosis, recent studies have suggested targeting macrophage activation as a way to ameliorate fibrosis and associated autoimmune renal diseases [30].

## 3. Changes in the Renal Microenvironment

ECM accumulation and remodeling are key characteristics of renal fibrosis and major drivers of end-stage CKD. The following section discusses these major changes during renal fibrosis, including not only changes in the ECM composition but also changes in the organization of the ECM, changes in the ECM pH that affect the structure and function of the renal compartments, and responding changes in the polarity of cells embedded in the ECM.

### 3.1. Changes in ECM Composition in Renal Fibrosis

The ECM is an important structural support and regulator of cell behavior in the kidney. The overall composition can be broken down into two primary structures: the basement membrane and the interstitial matrix [5]. The basement membrane serves as a barrier for epithelial and endothelial cells, and is primarily composed of the ECM proteins laminin and Col IV. The surrounding interstitial matrix serves as scaffolding for mesenchymal cells such as fibroblasts to move within. In renal fibrosis, activation of myofibroblasts, partial EMT, and partial EndoMT induce significant changes in the overall ECM composition (summarized in Table 1). Maintained activation of myofibroblasts creates a positive feedback loop that stimulates accumulation of ECM components, including fibronectin, glycosaminoglycans, and versicans, that continues to alter the original renal structure [8,13]. Increased synthesis of the ECM protein fibronectin in particular is a major marker of fibrotic ECM [31]. Fibronectin synthesis is stimulated by fibrotic cytokines like transforming growth factor-beta (TGF-*β*) and induces myofibroblast differentiation, partial EMT and EndoMT [31–33]. Progression of EMT and EndoMT can then in turn upregulate fibronectin and Col I and III synthesis. In addition to upregulated expression of fibronectin in myofibroblasts and macrophages, mutations of the gene can also contribute to CKD progression: inherited mutations drive fibronectin glomerulopathy and lead to abnormal deposition of fibronectin in the tubular interstitium that can progress to end stage renal failure [34,35]. Another rare renal disease associated with excess ECM deposition is Col III glomerulopathy. This is characterized by increased deposition of Col III in the glomerulus, which results in proteinuria, hypertension, and renal failure [5]. The prominent role of fibronectin and Col synthesis in driving this profibrotic environment suggests that they may serve as potential therapeutic targets.

### 3.2. Changes in ECM Organization and Mechanics in Renal Fibrosis

In addition to changes in ECM composition, renal fibrosis also affects the structural organization and mechanical properties of the ECM [36]. MMPs are major regulators of ECM remodeling through their proteolytic degradation of ECM proteins. Although MMPs have previously been viewed as antifibrotic due to their prominent role in degrading ECM proteins, increasing evidence suggests both an antifibrotic and profibrotic effect depending on their targeted ECM protein and the cell type responsible for expression [5,37]. Reduction of MMP activity increases accumulation of ECM proteins, contributing to increased basement membrane thickness and stiffening of the interstitial matrix [37]. However end-stage kidney disease and diabetic kidney diseases have shown significant upregulation of MMP-2 and MMP-9, respectively [38]. MMP-2 and MMP-9 drive kidney fibrosis by degrading the basement membrane proteins Col IV and laminin but also drive profibrotic events including EMT, EndoMT, and immune cell invasion [38]. Investigating the role that these MMPs have in regulating the renal microenvironment, and how disruptions in activity lead to renal fibrosis could identify new biomarkers and therapeutic measures.

Along with changes in ECM-related proteolytic activity, reorganization of ECM proteins is significantly altered in renal fibrosis. This again is most evident in changes to Col I and fibronectin. Fibronectin is secreted in a compact conformation [39], but when bound to integrins, cellular traction forces stretch it to form fibrils [40] (reviewed in [41,42]). In fibrosis, Col I and fibronectin are reorganized into aligned fibril tracts that increases the anisotropic stiffness of the microenvironment [36]. Inhibition of fibronectin fibril assembly using a 49-residue fragment of the *S. pyogenes* F1 adhesin protein reduced renal fibrosis in a UUO mouse model by preventing immune cell infiltration and Col I synthesis [43]. Application of the same recombinant peptide in a UIR (unilateral ischemia-reperfusion) mouse model demonstrated similar results (decreased immune cell infiltration and attenuated fibrosis) [32]. These studies suggest that targeting ECM remodeling may serve as a potential therapeutic avenue to prevent fibrosis.

Remodeling of the ECM in renal fibrosis alters the mechanical properties of the ECM. The assembly of fibronectin into fibrils drastically stiffens the ECM microenvironment [44,45]. These changes in stiffness are a key hallmark of fibrosis: fibrotic renal tissue is significantly stiffer than healthy kidney tissue [36,46]. Increased ECM stiffness can induce fibrosis in otherwise healthy tissue, indicating that ECM mechanics and organization affect cell behavior independent of cytokine activity [18]. Altered mechanotransduction in response to surrounding ECM remodeling has been shown to drive tubular atrophy, where tubule epithelial cells exhibit an elongated phenotype with decreased tight junctions and decreased cadherin expression [8].

Increased tissue stiffness also alters mechanosensitive signaling by altering the presentation of cytokines to cells by exposing cryptic binding sites for cytokines in ECM proteins, promoting fibrotic behaviors such as myofibroblast differentiation, partial EMT and partial EndoMT [15]. Additionally, increased tissue stiffness upregulates MMP synthesis, which in turn promotes ROS synthesis and EMT [5]. It can also be associated with the increased invasion of immune cells into the tubulointerstitium and glomerulus [27]. Due to its role in promoting chronic fibrosis, targeting specific cell–ECM interactions that drive increased tissue stiffness has been investigated for a number of fibrotic diseases [32].

### 3.3. Altered pH in Renal Fibrosis

Cellular function is affected by the pH of the surrounding microenvironment [47]. Lactate dehydrogenase (LDH) is the enzyme responsible for converting pyruvate into lactate, the conjugate base of lactic acid. An increase in LDH in renal tissue leads to an increase in lactic acid concentration, resulting in the decreased pH of the environment. LDH is stored in the cytoplasm, so small amounts of tissue damage can drastically alter the LDH levels of the tissue [48]. Lactic acid is an important marker of fibrosis and increases TGF-*β* activity in multiple fibrotic diseases [49]. As lactic acid increases in renal tissue, increased basement membrane remodeling occurs. Most notably, Col IV and VII are broken down by increased concentrations of collagenase and cathepsin B, and are replaced by Col I [50]. Due to the role of TGF-*β* in fibrosis, the role of LDH levels could be identified as a possible early biomarker of fibrosis.

### 3.4. Altered Extracellular Glucose in Renal Fibrosis

The kidney plays a major role in regulating blood glucose levels via reabsorption in the proximal convoluted tubules and gluconeogenesis and glycolysis in the tubules [5]. Epithelial cells of the proximal tubule use sodium-glucose co-transporters (SGLTs) and glucose transporters (GLUTs) to uptake glucose filtered from blood. In cases where extracellular glucose is high, hyperglycemia induces hypertrophy and apoptosis of TECs and mediates signaling pathways that exacerbate fibrosis such as TGF-*β* [51]. Hyperglycemia facilitates increased *O*_2_− production and ROS-mediated oxidative stress [52]. Low ROS levels are necessary to maintain cellular function in survival, proliferation, and growth [47]. However, a buildup of ROS in the cell can lead to inflammation and eventual cell death. Increased recruitment of pro-inflammatory cells forms a positive feedback loop of fibrotic progression by increasing the deposition of ECM components.

Targeting extracellular glucose and glucose-related pathways has provided approved therapeutics for renal fibrosis. Metformin is a commonly prescribed drug to treat Type-2 diabetes through the activation of AMPK (AMP-activated protein kinase), which, in hyperglycemic conditions, ameliorates autophagy and EMT in TECs and significantly decreases accumulation of ECM proteins such as Col and fibronectin [53]. SGLT2 inhibitors have been widely studied as a promising therapeutic [54]: dapagliflozin (farxiga) is an FDA approved therapeutic for CKD that inhibits SGLT2 function and blocks reabsorption of glucose into cells [51,54]. These successful therapies show that targeting glucose and metabolic processes ameliorates fibrosis progression.

### 3.5. Disrupted Apicobasal Polarity in Renal Fibrosis

Polarity is particularly important in kidney function, as the movement and reabsorption of nutrients depend on the proper localization of channels, transporters, junctional proteins, and ECM binding proteins to either the lumenal or interstitial side of the nephron. UUO-induced fibrosis indicates that there is a change in apicobasal polarity in both the glomerulus and tubules [4]. Increased EMT and myofibroblast differentiation impacts cell signaling. TGF-*β* itself can bind to PAR3 and PAR6 to promote the destabilization of tight junctions [55]. In addition, TGF-*β* promotes EMT, resulting in abnormal cell division and increasing the diameter of tubules [56]. EMT transcription factors Snail and Zeb1 bind to and repress promoters of Crumbs3 and other protein complexes related to polarity [57]. Disrupted polarity can lead to cell infiltration into the glomeruli and tubular interstitium resulting in increased fluid uptake due to ATPases transporting into the lumen instead of out [58]. Considering the major role of EMT and myofibroblast differentiation in disrupting the polarity of the nephron, identification of the mechanisms driving these cellular processes is a promising target for preventing renal fibrosis and overall renal failure.

## 4. Cell–ECM Bi-Directional Signaling: Integrins and Extracellular Vesicles

Having reviewed both cell and ECM-related changes involved in renal fibrosis, we now turn our attention to the direct and indirect ways in which cells and the ECM interact, focusing on two key areas: the role of integrin-mediated signaling and the role of extracellular vesicles. We will begin this section with a discussion of integrin-mediated interactions, discussing the role of integrins in both transducing signals from the ECM and activating latent cytokines embedded in the ECM. We will then focus on the role of extracellular vesicles, which provide a mechanism for cells to secrete and transmit new ECM components to both neighboring cells and directly to the ECM.

### 4.1. Integrin-Mediated Signaling

Interactions between cells and the surrounding ECM are mediated by cellular adhesion molecules including selectins, immunoglobulins, cadherins, and integrins. Of particular relevance to renal fibrosis are integrins, which are heterodimeric glycoproteins composed of an *α* and *β* monomer [59–61]. These monomers have transmembrane and cytoplasmic regions that connect the outside ECM to intracellular cytoskeleton components via focal adhesions and adaptor proteins [62]. Integrins are essential in cell traction force generation, allowing cells to sense ECM stiffness [44], migrate [44], and assemble ECM proteins into fibrous structures [63].

**Figure 2.**
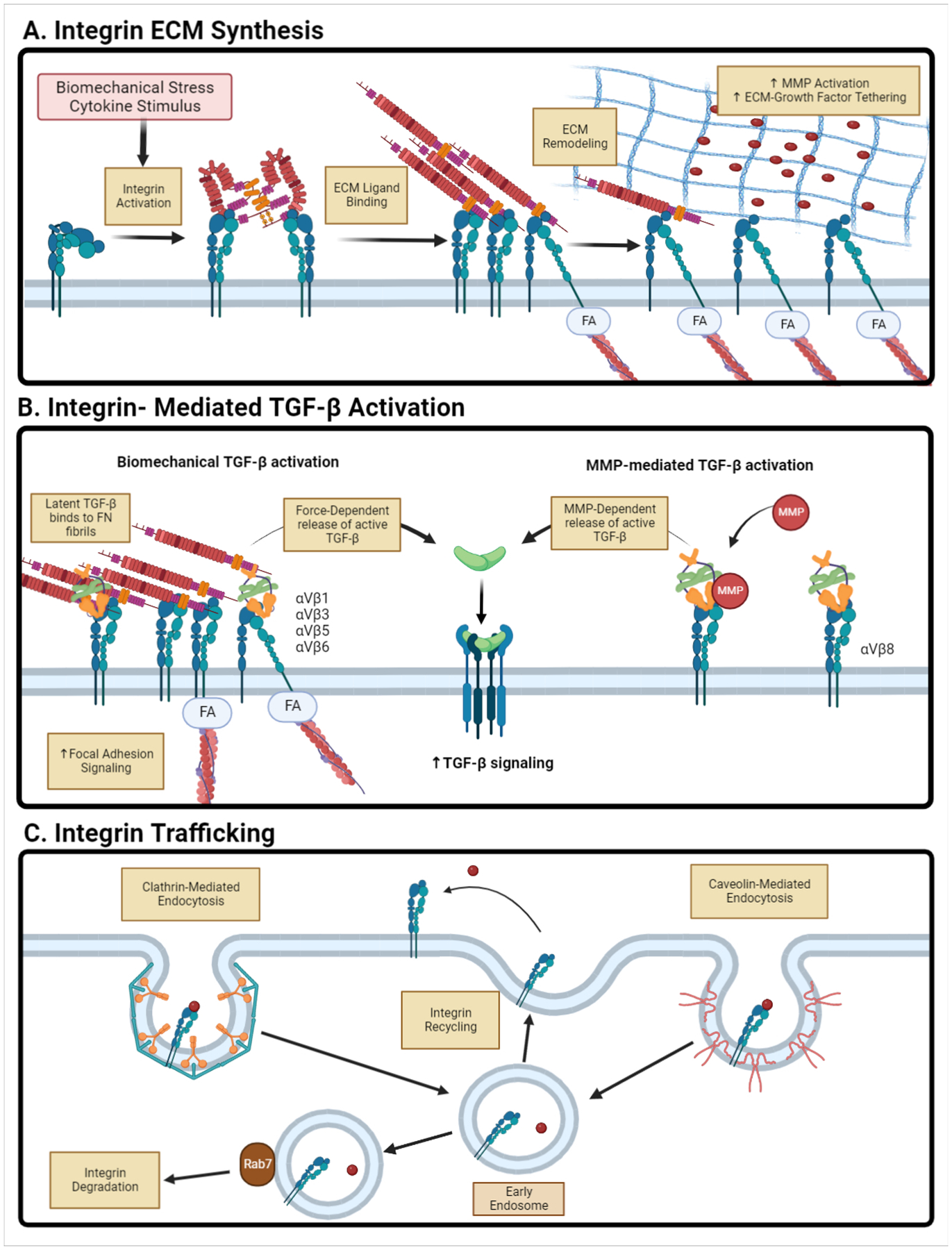
Schematic of integrin signaling in renal fibrosis. (A) ECM proteins act as integrin ligands that activate downstream signaling that promotes ECM synthesis and assembly. Increased ECM assembly opens tethering sites for growth factors and MMPs that further promote fibrogenesis. (B) Integrins are critical in regulating TGF-*β* signaling through the mechanical-dependent and MMP-dependent release of active TGF-*β* from LAP. (C) Integrin endocytosis mediates ECM signaling. Two main pathways are shown: clathrin-mediated and non-clathrin-mediated endocytosis. Renal fibrosis sees increased recycling of integrins back to the cell surface that promotes continued fibrotic signaling pathways. Figure generated using Biorender.com.

Ligand-specific binding to integrins mediates intracellular signaling processes. Many ECM proteins can bind to integrins and act as ligands to initiate cell behaviors including migration and proliferation (Figure 2). Specialized cells express distinct populations of integrins on their surface [64]. For example, podocytes express integrin *α*_3_*β*_1_ which binds to laminin, while mesangial cells express Col-specific binding integrins such as *α*_1_*β*_1_ and *α*_2_*β*_1_ [5]. Integrins play a number of different roles in maintaining kidney homeostasis. The loss or mutation of *α*_3_*β*_1_ integrins is characterized by severe kidney disease and overall tubulointerstitial fibrosis [64]. *α*_1_*β*_1_ integrin binds Col IV, which is a primary component of the tubular basement membrane. Deletion of *α*_1_*β*_1_ leads to exacerbation of glomerulosclerosis via increased Col-degrading MMP activity, increased ROS production, and decreased tyrosine phosphorylation of profibrotic cytokine receptors such as TGF-*β*RII [63]. On the other hand, knockouts of integrins that bind to interstitial matrix proteins such as Col I and fibronectin resulted in reduced fibrotic activity in UUO renal fibrotic mouse models [5,65]. *α*_2_*β*_1_ deletion in mice ameliorated kidney fibrosis in Alport syndrome mice, seen through a decrease in immune cell invasion and ECM deposition [63]. These studies have demonstrated that the loss of basement membrane-associated integrins results in exacerbated renal fibrosis, while the loss of interstitial matrix-associated integrins ameliorated fibrosis. Previous work has also shown that reducing cell–ECM signaling decreases myofibroblast activation and EMT progression [62].

### 4.2. Integrin-Mediated Latent TGF-β Activation

Along with the mediation of cell–ECM interactions, integrins are also critical in activating cytokines and growth factors tethered in the ECM. For example, the *α*_*v*_ integrin family activates latent TGF-*β* [66]. TGF-*β* is synthesized in a latent state, encased within the latency-associated peptide (LAP) and bound to latent TGF-*β* binding protein (LTBP), which prevents TGF-*β* from binding to its receptors. The release of active TGF-*β* requires binding of the complex to integrin *α*_*v*_ via the RGD domain found on LAP [62,66]. Depending on the integrin dimer, TGF-*β* is released by different mechanisms: integrins *α*_*v*_*β*_1_/*β*_3_/*β*_5_ (myofibroblasts/fibroblasts) and *α*_*v*_*β*_6_ (epithelial cells) bind to LAP and release active TGF-*β* via mechanical stretch [66], while *α*_*v*_*β*_8_ binds to LAP and releases TGF-*β* through the recruitment of MMPs [66]. This increased concentration of active TGF-*β* in the environment can readily bind to TGF-*β* receptors on the cell surface [65,66].

### 4.3. Integrin Trafficking and Endocytosis

Given the prominent role of integrins in both signal transduction from the ECM and the activation of tethered growth factors, it is not surprising that integrin trafficking to the plasma membrane is a critical regulator of ECM signaling. There are two primary methods of surface protein endocytosis: clathrin-mediated [67] and non-clathrin-mediated such as caveolin-1 [68]. Endocytosis is an important regulator of not only integrin and surface receptor activity, but also regulates surrounding ECM structure. Previous work has shown that caveolin-1-dependent endocytosis of integrin *β*1 regulates fibronectin degradation at the cell surface [69].

A significant upregulation of caveolin-1 expression is seen in rodent models of CKD and diabetic nephropathy, suggesting that the dysregulation of endocytosis mechanisms plays a role in maintaining fibrotic cell phenotypes and environment [68]. High extracellular glucose concentration, commonly observed in diabetic nephropathy, dysregulates the endocytosis of integrins and other cell-surface receptors [70]. Expression of these endocytotic factors are also implicated in regulation of MMP signaling in renal fibrosis [71,72]. Studies in UUO mouse models showed that inducing Rab7 expression initially suppresses renal fibrosis but then promotes its progression due to induced autophagy [73].

### 4.4. Therapeutic Targeting of Integrins

Given the major role that integrins play in mediating fibrotic phenotypes via cell–ECM signal transduction, ECM assembly, and cytokine activity, there have been significant efforts to therapeutically target integrin activity [65]. Although there have been a number of therapeutics that have gone through clinical trials, results have been varied. A lack of kidney-specific biomarkers has made it difficult to determine the clinical efficacy of treatments [65]. The review by Slack et al. provides a thorough discussion of clinical trials aimed at targeting integrins [65]. One potential reason for poor clinical trial efficacy is that, in addition to their role in renal fibrosis, integrins are critical mediators of development and overall renal function. For example, while integrins such as *α*_1_*β*_1_ exacerbate fibrosis, it is also a regulator of renal development and normal regenerative mechanisms [63]. Targeting integrins therapeutically most likely requires more in-depth studies of cell-specific knockouts to identify specific targets and improve delivery methods to prevent damage to healthy tissues.

### 4.5. Extracellular Vesicles

Extracellular vesicles (EVs) are small plasma membrane-encased particles. EVs are able to transport proteins, receptors, DNA, and RNA to mediate cell–cell communication. These can be further broken into two classes: exosomes and microvesicles [74,75]. Exosomes range from 40 to 150 nm and are derived from endosomal compartments. Microvesicles are larger than exosomes and are derived from the budding of the plasma membrane on the cell surface. In a healthy renal system, EVs derived outside the kidney cannot pass through the Bowman’s Capsule [76]. Due to this, EVs in healthy kidneys are solely derived from renal cells such as TECs and endothelial cells. An increase in EV production and secretion occurs in response to fibrotic injury [74]. These EVs contain higher populations of pro-fibrotic molecules such as TGF-*β*1. These EVs are important for paracrine signaling throughout the kidney and are able to travel through tubules to reach proximal cells in the nephron. Exosomes collected from high-glucose treated macrophages promoted TGF-*β*-induced activation of mesangial cells [77]. Knockdown of TGF-*β* signaling was able to ameliorate these changes [77]. Although there is clear evidence of the pro-fibrotic role of EVs, other work has suggested a possible therapeutic role of EVs. The use of urine-derived EVs from healthy patients had therapeutic effects in decreasing fibrosis progression [76,78].

Another interesting aspect of paracrine signaling using EVs is the ability of EVs to function as dynamic structural and functional components of the ECM and regulators of ECM behavior. Work from Chanda et al. investigated the role of EVs in idiopathic pulmonary fibrosis and showed that EVs carried the ECM protein fibronectin on the surface [79]. Exposing fibroblasts to exogenous EVs containing profibrotic factors activates fibrotic behaviors such as myofibroblast differentiation and increased macrophage invasion [80,81]. This is an interesting indicator that EVs can possibly play a role in prolonged fibrotic signaling. EVs released from tubular epithelial cells directly bind to ECM proteins and stimulate myofibroblast differentiation and activity [74,82].

## 5. Relevant Signaling Pathways in Renal Fibrosis

**Figure 3.**
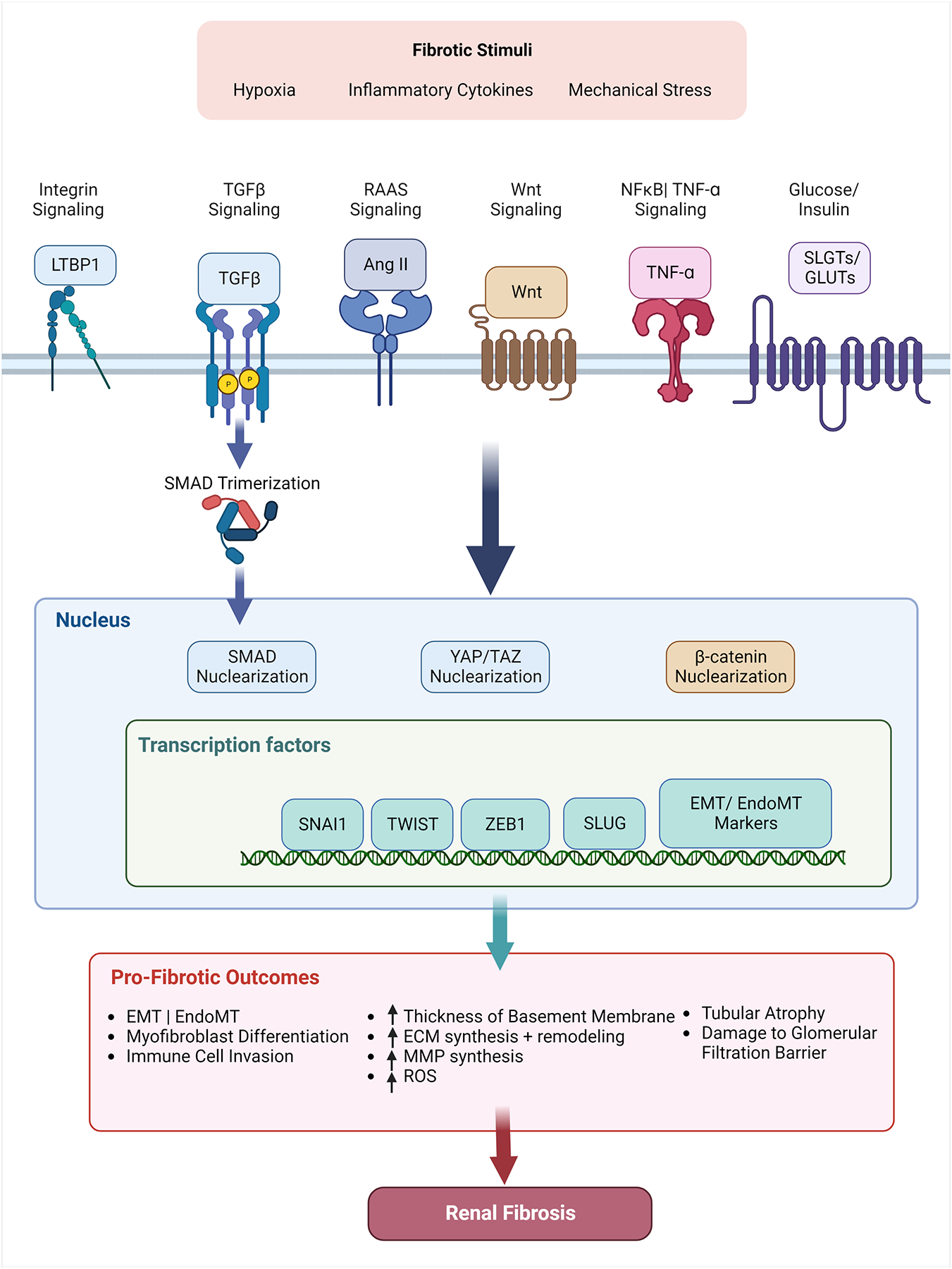
Relevant signaling pathways that drive renal fibrosis. Activation of pathways (Integrin, RAAS, TGF-*β*, Wnt, NF*κ*B, and Glucose) drive increased transcription of profibrotic factors such as Snail, Twist, Zeb1, and Slug. Increased expression drives a number of profibrotic outcomes such as EMT and EndoMT in epithelial cells and endothelial cells respectively. Figure generated using Biorender.com.

As discussed, renal fibrosis proceeds via bidirectional signaling between cells and the surrounding ECM: injury drives altered cell signal transduction that results in ECM morphological changes. These ECM changes in turn lead to further alterations in cell signal transduction. We conclude this review with a discussion of signaling molecules that have been implicated in renal fibrosis, including proteins that drive ECM alterations in response to inflammatory signals and proteins that respond to altered ECM signaling to further aggravate renal fibrosis as shown in Figure 3. We will also discuss attempts to target these pathways therapeutically.

### 5.1. TGF-β Superfamily

The TGF-*β* superfamily includes the three isoforms of TGF-*β*, the seven isoforms of bone morphogenetic protein (BMP), and activins [83]. TGF-*β* and BMP act as ligands for serine and threonine kinase receptors on the cell surface. When bound, the receptors form tetrameric complexes, where TGF-*β* receptor II (TGF-*β*RII) phosphorylates TGF-*β* receptor I (TGF-*β*RI). Activated TGF-*β*RI phosphorylates downstream Smad signaling, and are then shuttled to the nucleus to drive gene transcription [83,84]. TGF-*β* isoforms drive phosphorylation of Smad2/3, while BMP isoforms drive phosphorylation of Smad 1/5/8 [85].

Increased activation of in TGF-*β*1 is observed in renal fibrosis [86]. This upregulation of TGF-*β*1 activity is induced partly by changes in ECM composition and stiffness. As stated before, TGF-*β*1 is secreted in a latent state, bound by LAP. Increased fibronectin fibril formation increases TGF-*β*1 release from its latent state and colocalization at the cell surface at stated in section 4.2, forming a positive feedback loop that sustains fibrotic signaling [43,87]. TGF-*β*1 binding to TGF-*β*1 receptors activates Smad 2/3 that then form a trimeric complex with Smad 4. The trimeric Smad complex is then translocated to the nucleus and regulates transcription of fibrogenic genes that promote myofibroblast differentiation, EMT, and EndoMT [88]. Previous work has shown that Smad 3 is fibrogenic and highly upregulated in diabetic nephropathy, hypertensive nephropathy, and aristolochic acid-induced nephropathy [55]. Deletion of Smad 4 was able to inhibit fibrosis by suppressing Smad 3 induced upregulation of miRNAs that are critical regulators of TGF-*β* signaling such as miRNA-200 and miRNA-34, which are both known regulators of Snail and Zeb1 activation that promote EMT and EndoMT [55,85].

BMP7, another member of the TGF-*β* superfamily, has previously been shown to have anti-fibrotic effects through counter-regulation of the TGF-*β*/Smad3 signaling pathway [55,86]. BMP activates Smad 1/5/8 signaling that has been shown to be renoprotective through the inhibition of CTGF expression, and increased Smad6 transcription that blocks receptor phosphorylation [88]. In healthy kidneys, TGF-*β* and BMP counter-regulate each other through downstream Smad activation. In addition, TGF-*β*/Smad3 signaling itself also promotes the transcription of Smad7, which inhibits Smad3 activity itself. However, in response to increased TGF-*β* synthesis and profibrotic signaling, these regulatory feedback loops between TGF-*β* and BMP are lost. The upregulation of TGF-*β*/Smad3 signaling leads to the increased ubiquitin-proteasome degradation of Smad7, as well as the inhibition of the BMP signaling pathway, which further promotes profibrotic signaling events [86].

While TGF-*β*1 is the most-studied isoform of TGF-*β* in renal fibrosis and CKD, the other isoforms have also been implicated in maintaining the fibrotic environment. TGF-*β*2 and TGF-*β*3 have both been shown to be profibrotic, similar to TGF-*β*1 [89]. Although compared to TGF-*β*1, TGF-*β*2 binds to TGF-*β* receptors at a lower affinity, it is still able to activate downstream Smad signaling that promotes EMT, myofibroblast differentiation, and increased ECM deposition [89]. The role of TGF-*β*3 is still not fully understood. Although present in all renal cells, it is mainly expressed in podocytes along with TGF-*β*2 [89]. Heterozygous TGF-*β*3 knockout mice exhibit increased EMT, thickening of the glomerular basement membrane, and podocyte migration, suggesting an anti-fibrotic role of TGF-*β*3 [90]. This may be cell type-specific, since TGF-*β* isoform signaling is highly dependent on receptor expression, co-receptor expression, and isoform expression levels [91].

Despite the prominent role of TGF-*β* signaling in renal fibrosis, clinical trials that target TGF-*β*1 activation have had mixed results. Inhibition of TGF-*β*1 activity using a blocking antibody (LY2382770) did not improve renal function in patients with diabetic nephropathy [92]. In contrast, Pirfenidone, which inhibits all three isoforms of TGF-*β*, resulted in improved eGFR in patients with pulmonary fibrosis [93]; current clinical trials using Pirfenidone to treat CKD are ongoing (NCT04258397). Though TGF-*β* is a prominent promoter of fibrosis, it also is active in maintaining homeostasis. Deficiency of TGF-*β*1 in mice results in lethal inflammation and early death [55]. Due to its close tie with homeostatic bodily functions, instead of directly targeting TGF-*β*, many recent studies have instead directed attention to downstream profibrotic molecules such as the Smads or miRNA regulators of the pathway as possible therapeutic targets [55,94].

### 5.2. Wnt/β-Catenin

The Wnt family is composed of lipid-modified glycoproteins that have critical roles in regulating nephrogenesis [18]. Wnt binding induces accumulation of the cofactor *β*-catenin that regulates downstream mediators of renal fibrosis such as TGF-*β*, NF*κ*B, and Snail [18,95]. Chronic activation of the Wnt/*β*-catenin pathway promotes profibrotic events like EMT, immune cell invasion, and myofibroblast differentiation [18,96]. Downstream *β*-catenin signaling induces increased synthesis of MMP-7 in multiple renal diseases [95]. Upregulation of MMP-7 forms a positive feedback loop that continues to elevate *β*-catenin and MMP-7 levels. Inhibition of this signaling pathway has been cited as a possible therapeutic target for renal fibrosis [95]. However, like TGF-*β* signaling, there is more research needed to fully understand the mechanisms of activation to identify therapeutic targets in the pathway.

### 5.3. NFκB/TNF-α

NF*κ*B is a family of transcription factors that regulate immune and inflammatory responses. One of the main roles of NF*κ*B in acute injury is the recruitment of immune cells such as monocytes, macrophages, and neutrophils that secrete inflammatory cytokines to promote myofibroblast activation and EMT [97]. In its inactive state, NF*κ*B is kept in the cytoplasm and bound to I*κ*B to prevent nuclear translocation. In response to a trigger, I*κ*B Kinase is activated and phosphorylates I*κ*B to release NF*κ*B. These can translate into the nucleus and promote pro-inflammatory gene expressions and increased MMP-9 production. Increases in Ang II, TGF-*β*, and other inflammatory stimulators promote upregulation of NF*κ*B [92,98]. The progression of fibrosis is driven by chronic crosstalk between these signaling pathways that maintains activation of myofibroblasts and ECM deposition. Inhibition of NF*κ*B pathway reduces inflammation and fibrosis in UUO and AKI models [98].

### 5.4. Angiotensin II/RAAS Pathway

The renin-angiotensin-aldosterone system (RAAS) regulates water and sodium content in the kidney. The RAAS signaling pathway effects multiple compartments of the renal system. In terms of the vasculature, RAAS stimulates the release of the hormone Vasopressin which is involved in vascular constriction and permeability of the collecting duct [2]. Rats chronically infused with Ang II develop renal injuries including tubular atrophy and dilation, interstitial monocyte infiltration, and excessive col IV deposition [99]. Increased angiotensin II (Ang II) promotes synthesis of other pro-fibrotic signals such as TGF-*β*, NF*κ*B, Wnt, and CTGF [100,101]. In addition to the upregulation of TGF-*β* directly, Ang II also stimulates Smad signaling independently, further activating downstream effects such as increased Col and fibronectin synthesis.

Dysregulation of the RAAS pathway promotes renal fibrosis through the direct promotion of profibrotic cell activities: myofibroblast differentiation, ECM synthesis, immune cell invasion. Inhibition of the RAAS signaling pathway using Ang II receptor blockers is a common treatment for cardiac and renal diseases [7,99,101]. Improving our understanding of crosstalk between RAAS and other profibrotic signaling pathways will help identify potential ways to attenuate fibrosis-induced kidney damage.

### 5.5. Transcription Factors + Coactivators

#### 5.5.1. Transcription Factors

The transcription factors Snail and Twist1 both induce partial EMT in tubular epithelial cells [102,103]. Increased expression of Snail and Twist1 promote p21-mediated G2/M cell cycle arrest in TECs, leading to the dilation of tubules [4,21]. Activation of this partial EMT state also promotes cell detachment from ECM that leads to the dysregulation of TEC transport function (decreased expression of aquaporins and *Na*^+^/*K*^+^ pumps) [57]. Snail directly binds to Crumbs3 and claudin proximal promoters to downregulate expression, thus leading to the loss of apicobasal markers [57]. Deletion or silencing of these transcription factors in mice showed a decrease in EMT progression and ameliorate that loss of tubular structure [21]. Zeb1 is another transcription factor associated with EMT progression through the activation of the TGF-*β*1 signaling pathway [4,104]. Deletion or silencing of Zeb1 or Snail in mice decreased EMT progression and maintained tubular structure [4]. Overexpression of Snail induced increased profibrotic markers, inflammation, and myofibroblast accumulation [4]. These transcription factors are downstream targets of multiple profibrotic signaling pathways including TGF-*β*, Wnt, and NF-*κ*B, suggesting that the inhibition of their activity will prevent fibrotic behavior.

#### 5.5.2. Mechanosensitive Coactivators

YAP/TAZ are mechanosensitive co-activators that regulate cell–ECM mechanotransduction in response to changes in the surrounding microenvironment. Increases in ECM stiffness trigger YAP/TAZ nuclear transport in fibroblasts which promotes the production of ECM proteins and profibrotic signaling pathways such as TGF-*β* and Wnt [105,106]. The progression of EMT in epithelial cells disrupts polarity regulators such as Crumbs and drives YAP/TAZ nuclear localization, promoting increased ECM protein and cytokine synthesis [106]. Biopsies of patients with tubulointerstitial fibrosis exhibit high YAP/TAZ expression [106]. The treatment of UUO mice with the YAP inhibitor verteporfin reduced interstitial fibrosis through decreased myofibroblast differentiation and attenuated Col I and fibronectin accumulation [105]. Further investigation of myofibroblast-specific deletion of YAP/TAZ partially reversed UUO-induced fibrosis through decreased myofibroblast markers [105]. Overall, YAP/TAZ are implicated in maintaining the profibrotic renal environment by inducing myofibroblast differentiation and EMT. Inhibition of their activity is a promising therapeutic target that may not only prevent fibrosis, but also partially reverse it.

## 6. Future Therapeutic Directions

While there have been some successes in treating CKD therapeutically, there are still limitations to each that prevent successful treatments for all CKD patients. The underlying difficulty in treating renal fibrosis and CKD is caused by the numerous factors and underlying conditions that drive the profibrotic microenvironment. Another major limitation in targeting renal fibrosis is the translation of research from preclinical models to a clinical setting. An interesting example is TGF-*β*. In vitro research has shown that direct inhibition of TGF-*β* reduces profibrotic cell behaviors [88]. However, translation to in vivo and clinical trials failed to produce significant changes in renal fibrosis and overall CKD prevention [55,92]. Due to this, fibrosis therapeutics in vivo have shifted focus to targeting signaling pathways using small molecules and downstream signaling factors instead [55]. The development of patient-specific treatments is also a major field of study to tailor treatments of CKD driven by different underlying factors such as diabetes or autoimmune disorders. The use of patient-derived EVs has been proposed as a means of drug transport to reduce patient rejection and prevent off-target delivery. Prior work has shown that EVs can be tuned to mimic the cell–ECM interactions in healthy tissue to promote anti-fibrotic signaling [76]. In addition, the use of EVs as a non-invasive biomarker has been discussed as a new method for identifying early-stage renal fibrosis [77]. This is expected to improve the guidelines to validate preclinical study results and all for earlier diagnosis of CKD.

## 7. Conclusions

CKD is a significant clinical problem in the world, and much of the damage is caused by the presence of renal fibrosis, which is characterized by excessive ECM assembly that disrupts kidney function. This review has discussed the interactions between cells and their surrounding ECM, both in terms of how renal cell populations alter the ECM, and how the altered ECM in turn affects renal cells. We have discussed relevant cell populations, significant alterations to the ECM and microenvironment, the dynamics of cell–ECM interactions, and relevant signal transduction pathways that drive these changes.

Although the root cause for initiation of fibrosis may vary, the commonality that promotes chronic fibrosis stems from cell–ECM crosstalk. There is still a need to improve our understanding of the crosstalk between signaling pathways that promotes the profibrotic activity of different cell lines. As stated before, renal fibroblasts and pericytes are known to be the main population of myofibroblast progenitors, but there are also recent studies that indicate that macrophages and bone marrow-derived fibroblasts may also contribute to maintaining this population [11,27]. The transformation of renal epithelial and endothelial cells lines through EMT and EndoMT respectively contribute to ECM deposition and promote myofibroblast activation that disrupts the structural integrity and overall function of the renal compartments. By improving our understanding of the relative roles of each of these cells in renal fibrosis progression, we can hope to identify better targets that block the progression of renal fibrosis.

## Figures and Tables

**Table 1.** Changes in ECM composition during renal fibrosis. This table summarizes changes in ECM composition in the glomerulus and tubulointerstitium during the progression of renal fibrosis.

Component	Healthy Kidney	Fibrotic Kidney
Glomerulus	**Mesangial Matrix:** Col IV, V, FN	**Mesangial Matrix:** increase in Col IV, V, FN
	**Basement Membrane:** Col IV, I, III, VI, VII, XV, XVII	**Basement Membrane:** increase in Col IV, I, III, VI, VII, XV, XVII
	**Bowman’s Capsule:** Col IV	**Bowman’s Capsule:** increase in Col IV
Tubulointerstitium	**Basement Membrane:** Col IV	**Basement Membrane:** thickens, increase in Col IV
	**Interstitium:** Col I, II, III, V, VI, VII, XV, FN	**Interstitium:** Increase in Col I, II, III, V, VI, VII, FN

## Abbreviations

The following abbreviations are used in this manuscript:

*α*-SMA: *α*-Smooth Muscle Actin
AKI: Acute Kidney Injury
AMPK: AMP-Activated Protein Kinase
BMP: Bone Morphogenic Protein
CKD: Chronic Kidney Disease
CTGF: Connective Tissue Growth Factor
ECM: Extracellular Matrix
eGFR: estimated Glomerular Filtration Rate
EMT: Epithelial-Mesenchymal Transition
EndMT: Endothelial-Mesenchymal Transition
EPO: Erythropoeitin
GLUT: Glucose Transporter
LAP: Latency-Associated Peptide
LDH: Lactate Dehydrogenase
LTBP: Latent TGF-*β* Binding Protein
MMP: Matrix Metalloproteinase
MMT: Macrophage-Mesenchymal Transition
PDGFR*β*: Platelet-Derived Growth Factor Receptor-*β*
RAAS: Renin-Angiotensin System
ROS: Reactive Oxygen Species
SGLT: Sodium-Glucose Transporter
TEC: Tubular Epithelial Cells
TGF-*β*: Transforming Growth Factor-*β*
UIR: Unilateral Ischemia-Reperfusion
UUO: Unilateral Ureteral Obstruction
